# Impact of emergency medical helicopter transport directly to a university hospital trauma center on mortality of severe blunt trauma patients until discharge

**DOI:** 10.1186/cc11647

**Published:** 2012-09-28

**Authors:** Thibaut Desmettre, Jean-Michel Yeguiayan, Hervé Coadou, Claude Jacquot, Mathieu Raux, Benoit Vivien, Claude Martin, Claire Bonithon-Kopp, Marc Freysz

**Affiliations:** 1 Université de Franche Comté, CHU Besançon, Hôpital J Minjoz, Urgences/SAMU25, 25030, France; 2 Université de Bourgogne, CHU Dijon, Hôpital du Bocage, Département de Médecine d'Urgence, 21000, France; 3 Université de Lille II, CHRU Lille, Fédération des Urgences-SAMU59, 59000, France; 4 CHU Grenoble, Pôle Anesthésie-Réanimation, 38701, France; 5 Université Pierre et Marie Curie-Paris 6; GH Pitié-Salpêtrière, Assistance Publique-Hôpitaux de Paris Service d'Accueil des Urgences, 75013, France; 6 Assistance Publique-Hôpitaux de Paris, Hôpital Necker, SAMU de Paris, 75015, France; 7 Université de la Méditerranée, CHU Nord, Centre de traumatologie et Département d'Anesthésie Réanimation, 13915, France; 8 INSERM CIE 01, CHU Dijon, Centre d'Investigation Clinique-Epidémiologie Clinique, 21000, France

**Keywords:** severe trauma patients, helicopter transport, pre-hospital care, mortality

## Abstract

**Introduction:**

The benefits of transporting severely injured patients by helicopter remain controversial. This study aimed to analyze the impact on mortality of helicopter compared to ground transport directly from the scene to a University hospital trauma center.

**Methods:**

The French Intensive Care Research for Severe Trauma cohort study enrolled 2,703 patients with severe blunt trauma requiring admission to University hospital intensive care units within 72 hours. Pre-hospital and hospital clinical data, including the mode of transport, (helicopter (HMICU) versus ground (GMICU), both with medical teams), were recorded. The analysis was restricted to patients admitted directly from the scene to a University hospital trauma center. The main endpoint was mortality until ICU discharge.

**Results:**

Of the 1,958 patients analyzed, 74% were transported by GMICU, 26% by HMICU. Median injury severity score (ISS) was 26 (interquartile range (IQR) 19 to 34) for HMICU patients and 25 (IQR 18 to 34) for GMICU patients. Compared to GMICU, HMICU patients had a higher median time frame before hospital admission and were more intensively treated in the pre-hospital phase. Crude mortality until hospital discharge was the same regardless of pre-hospital mode of transport. After adjustment for initial status, the risk of death was significantly lower (odds ratio (OR): 0.68, 95% confidence interval (CI) 0.47 to 0.98, *P *= 0.035) for HMICU compared with GMICU. This result did not change after further adjustment for ISS and overall surgical procedures.

**Conclusions:**

This study suggests a beneficial impact of helicopter transport on mortality in severe blunt trauma. Whether this association could be due to better management in the pre-hospital phase needs to be more thoroughly assessed.

## Introduction

For severely injured patients, delayed control of hemorrhage is the main factor contributing to trauma mortality [1,2]. Direct access to a trauma center with definitive care reduces the risk of death [3]. Helicopter transport (HT) of the injured patient should improve access to the trauma center, but its use remains controversial [4]. Several studies have shown that trauma patients transported by helicopter are more severely injured, have longer transport times, and require more hospital resources than those transported by ground [5,6]. In recent large studies, HT was a predictor of survival compared with ground transport (GT) [7,8]. The debate about the factors that could explain the impact of HT on survival remains open [9]. This benefit could be attributed to a higher level of competence in helicopter crews or to improved care provided at the destination centers [10-12].

The French Intensive care Research in Severe Trauma (FIRST) is a multicenter cohort study of consecutive severe blunt trauma patients admitted to 14 University hospitals (UnivH) and ICUs within 72 hours of injury. A previous analysis demonstrated that pre-hospital medical management reduces 30-day mortality in severe blunt trauma compared with non-medical pre-hospital management [13].

The aim of this observational study was to evaluate the impact on mortality of injured patients within hospital discharge of HT versus GT directly from the scene to a UnivH trauma center.

## Materials and methods

### The French pre-hospital trauma rescue system

The French pre-hospital rescue system with the Mobile Intensive Care Unit (MICU) has been well described in the literature [13-15]. All MICUs are staffed by an emergency physician, a nurse, and a specially trained ambulance driver. The decision to use a helicopter (HMICU) is based on the suspected severity of the accident or trauma, distance from the trauma center, availability of the helicopter, and the suspected need for immediate recourse to a trauma center able to provide definitive care especially for specialized surgery. In France, ground MICUs (GMICUs) are staffed by a team from the closest hospital, while HMICUs are staffed by a team with an emergency physician from the regional University hospital. Both GMICUs and HMICUs can be dispatched simultaneously to the scene of the accident when necessary. The MICU may initiate early life-sustaining treatment at the scene of the accident and the patient is transported to the closest trauma center or to a UnivH trauma center [16,17]. When HMICU is dispatched to the scene of the accident, the injured patient is always transported by helicopter. The response to treatment and suspected severity is used by the dispatching physician to identify the most appropriate center for the patient [18]. Although there is still no formal certification process as described in some other Trauma Systems, the University hospitals can provide care in the same way as level one trauma centers in the United States, and are identified by the dispatching center as a regional trauma center.

## Patients

Between December 2004 and March 2007, the data on consecutive patients with severe blunt trauma were prospectively recorded in 14 UnivH in France. Inclusion criteria were age 18 or above and suspected severe blunt trauma, defined as trauma requiring admission to a UnivH ICU within 72 hours of injury or, in the case of early death before ICU admission, trauma managed by the MICU of a UnivH. Exclusion criteria were penetrating trauma and death occurring before any advanced life-sustaining treatment was administered. A total of 3,205 patients were eligible for inclusion in the FIRST study [13].

### Data collection

The eligibility criteria were checked online by the research assistants of the Coordination Center in Dijon (France). Every month, data were extracted by the Coordination Center for quality control. For missing, aberrant or illogical mandatory data, queries were sent to local research assistants. At the end of the inclusion period, data monitoring was performed by the Coordination Center to validate data quality on a random sample of 7% of patients. Unreliable variables were discarded from the analysis. The following data were collected: patient characteristics, data about the circumstances of the accident, condition of victims in traffic-related accidents, and rescue services mobilized for patient transport (ground or helicopter), hospital units involved in early care of the patient before admission to the ICU, clinical and biological data in the pre-hospital phase, at first hospital admission and at 24 hours and 72 hours after trauma, and clinical variables on patient discharge or death including all surgical procedures within the first 24 hours and until discharge or death within 30 days. During the pre-hospital phase, the following data were recorded: prehospital time defined as the time between the accident or the first call to the dispatch center and the hospital admission, initial physiological variables (systolic blood pressure (SBP), pulse oximetry (SpO_2_)), pupil status, Glasgow Coma Scale (GCS) and life-sustaining treatments (venous line, fluid loading and catecholamine administration, tracheal intubation, ventilation, blood products, and chest tube insertion). The accident was considered potentially severe if, in the case of a road traffic accident, at least one of the following was present: pedestrian, no safety equipment (air bag, seat belt, crash helmet, and so on), excessive speed, victim ejected/crushed/burned/cut free from the vehicle, death of other victims in the vehicle, vehicle fall of more than six meters. For the other accidents, the potential severity was defined as a fall of more than six meters, or crushing by farm equipment. The accident was considered to have occurred in the daytime if it occurred between 8:30 am and 6:30 pm and at the weekend between 1:00 pm on Saturday and 8:00 am on Monday. The trauma was suspected to be serious if, on the initial medical examination, there was a suspicion of fractured skull or flail chest, spine injury or, in the presence of limb amputation, severe burns, smoke inhalation or mydriasis. Data were collected on hemostatic procedures including arterial embolization and hemostatic thoracotomy or abdominal laparotomy, as well as orthopedic procedures including all types of bone fixation of the upper and lower limbs. For all patients, information on vital parameters and life-sustaining treatments was also collected upon arrival at the trauma center and 24 hours and 72 hours after the injury. Data were collected by ICU physicians and research assistants from the medical records of MICUs, emergency units and ICUs. The Abbreviated Injury Scale (AIS) was calculated according to the 1998 updated classification using medical, radiological and surgical reports. All problematic cases were reviewed by local ICU physicians.

The pre-hospital treatment was considered aggressive if at least three of the six life-sustaining treatments were administered during pre-hospital management (intubation, colloid and/or hypertonic saline solution infusion, continuous catecholamine infusion, pneumothorax aspiration or chest tube insertion, blood product administration and more than 1,000 ml of crystalloid infusion). All surgical procedures performed until ICU discharge were recorded and coded by physicians at the Coordination Center.

On patient discharge from the ICU or death (within 30 days), anatomic injury diagnoses with the corresponding AIS codes, and the ISS were collected [19-21].

According to French law (law 88-1138 relative to Biomedical Research of 20 December 1988 modified on 9 August 2004), this non-interventional study did not require approval by an Ethics Committee nor informed signed consent from the patients. The study was declared to, and approved by the French National Commission for Data Processing and Civil Liberties (authorization number 05-1059 obtained on 24 February 2005).

### End points

The main outcome measurement was the vital status at 30 days or at ICU discharge, if discharge occurred within the first 30 days.

### Statistical methods

Given their non-Gaussian distribution, quantitative variables were *a priori *categorized as follows: GCS score (<8, 8 to 13, >13), ISS (<15, 15 to 24, 25 to 34, ≥35), systolic arterial blood pressure (<90, 90 to 110, >110 mmHg), and SpO_2 _(<90%, ≥90 %). Descriptive characteristics were expressed as percentages, or means with standard deviations (SD), or medians with IQR. Univariate comparisons between groups (HT versus GT) were performed using chi-square tests or Fisher exact tests, when appropriate, for qualitative variables, and using the Wilcoxon test for quantitative variables.

A multivariate analysis was performed using logistic regression stratified on the center, where the outcome (30-day mortality) was introduced as the dependent variable. Independent variables included mode of transport (ground or helicopter) and all pre-hospital covariables associated either with the mode of transport or with 30-day mortality with a *P *value <0.20 in a bivariate analysis (model 1). For the covariable selection, we used a stepwise procedure excluding covariables with a *P *value greater than 0.10. A similar analysis strategy was used for further models that also included the ISS (model 2) and both the ISS and overall surgical procedures (model 3). Interaction terms between mode of transport and other independent variables were systematically tested. As none were significant, they were dropped from the final models. The Hosmer-Lemeshow test was used to check the models' goodness-of-fit (the *P *value was 0.33 for final model 1, 0.62 for final model 2 and 0.82 for final model 3). The discriminatory power of the models was quantified by the concordance index (C-index) corresponding to the area under the receiver operating characteristic (ROC) curve (C-index was 85.6% for final model 1, 87.7% for final model 2 and 88.2% for final model 3). The significance level was *P *<0.05. The statistical analyses were performed with SAS™ version 9.3 (SAS Institute Inc, Cary, NC).

## Results

**Figure 1:**
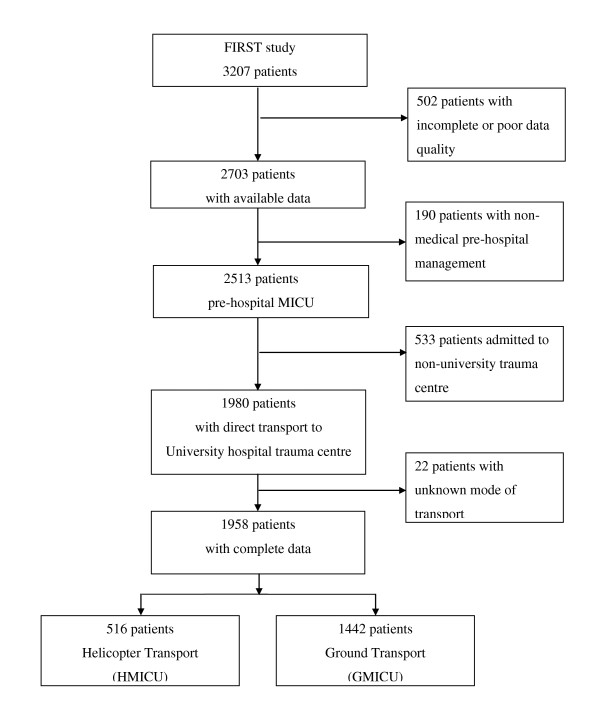
**Flow chart of the FIRST study and pre-hospital mode of transport**. FIRST, French Intensive care Research for Severe Trauma; GMICU, ground mobile intensive care unit; HMICU, helicopter mobile intensive care unit; MICU, mobile intensive care unit.

Of the 1,958 patients directly admitted to a University trauma center with complete data, 74% were transported by ground and 26% by helicopter (Figure 1). Patient characteristics and accident circumstances are given in Table 1. A suspicion of severe trauma was significantly more frequent for HMICU than for GMICU. The median time to hospital admission was higher for HMICU than for GMICU (2.3 hours, IQR 1.8 to 3.0 versus 1.8 hours, IQR 1.3 to 2.3, *P *<0.0001).

**Table 1:** Patient characteristics and accident circumstances of patients with severe blunt trauma according to mode of transport.

		Transport modality, number (%)		
	**All patients**, **number (%)** **number = 1,958**	**HMICU** **number (%)** **number = 516**	**GMICU** **number (%)** **number = 1,442**	***P***

**Age (years)**				
median (IQR) mean (SD)	37.0 (24.9 to 52.8) 40.6 (18.0)	39.2 (25.1 to 52.9) 40.9 (16.9)	36.0 (24.8 to 52.8) 40.6 (18.4)	0.33*
**Sex**				
men	1,483 (75.7)	395 (76.5)	1,088 (75.5)	0.62
women	475 (24.3)	121 (23.5)	354 (24.5)	
**Accident severity^a^**				
Yes	1,278 (67.2)	317(63.9)	961 (68.3)	0.073
No	680 (32.8)	199 (36.1)	481 (31.7)	
**Suspected trauma severity^b^**				
yes	994 (50.8)	286 (55.4)	708 (49.1)	0.014
no	964 (49.2)	230 (44.6)	734 (50.9)	
**Accident time**				
8:00 am to 6:30 pm	1,399 (72.5)	450 (87.7)	949 (67.0)	<0.001
**Accident day**				
week	1,366 (70.8)	336 (65.5)	1,030 (72.7)	0.002
weekend	563 (29.2)	177 (34.5)	386 (27.3)	
**Time to hospital admission (hours)**	number = 1,879	number = 507	number = 1,372	
median (IQR)	1.9 (1.4 to 2.5)	2.3 (1.8 to 3.0)	1.8 (1.3 to 2.3)	<0.001*
mean (SD)	2.4 (5.0)	3.1 (8.1)	2.1 (3.2)	

^a^Accident severity was defined if at least one of the following criteria was present: pedestrian; no safety equipment (air bag, seat belt, crash helmet, and so on); high velocity; victim ejected/crushed/burned/or cut free from the vehicle; death of another person in the same vehicle; fall of more than six meters. ^b^Trauma severity was suspected if at least one of the following criteria was present: suspicion of skull fracture, pelvic fracture, flail chest, spine injury, limb amputation, severe burns, smoke inhalation, mydriasis. (*) Wilcoxon Test. GMICU, ground transport of mobile intensive care unit; HMICU, helicopter transport of mobile intensive care unit.

Initial pre-hospital assessment and injury severity according to mode of transport are presented in Table 2. The proportion of patients with SBP lower than 90 mmHg was significantly higher in the HMICU group than in the GMICU group, as was the percentage of patients with severe spinal injury. The median ISS was 25 (IQR 18 to 34) for all patients, 26 (IQR 19 to 34) for the HMICU group and 25 (IQR 18 to 34) for the GMICU group. No differences in ISS were noted between the HMICU and GMICU groups.

**Table 2:** Initial assessment and Injury Severity Score according to mode of transport.

		Mode of Transport; number (%)		
	**all patients; number (%)** **number = 1,958**	**HMICU** **number = 516**	**GMICU** **number = 1,442**	***P***

**GCS **				
≥14	892 (46.0)	224 (44.0)	668 (46.8)	0.39
8 to 13	393 (20.3)	101 (19.8)	292 (20.4)	
<8	653 (33.7)	184 (36.2)	469 (32.8)	
**Abnormal pupils**				
No	1,474 (76.5)	386 (75.8)	1,088 (76.7)	0.71
Yes	454 (23.5)	123 (24.2)	331 (23.3)	
**SBP (mmHg)**				
≥110	1,324 (68.3)	329 (65.0)	995 (69.5)	0.03
90 to 110	344 (17.8)	89 (17.6)	255 (17.8)	
<90	270 (13.9)	88 (17.4)	182 (12.7)	
**SpO_2 _(%)**				
≥90	1,626 (84.8)	419 (84.5)	1,207 (84.9)	0.83
<90	292 (15.2)	77 (15.5)	215 (15.1)	
**AIS**				
head AIS ≥4	81 (41.4)	19 (38.6)	61 (42.4)	0.14
face AIS ≥4	1 (0.7)	7 (1.4)	6 (0.4)	0.05*
neck AIS ≥4	9 (0.5)	2 (0.4)	7 (0.5)	1*
thorax AIS ≥4	567 (29.0)	134 (26.0)	433 (30.0)	0.08
abdominal AIS ≥4	137 (7.0)	36 (7.0)	101 (7.0)	0.98
spine AIS ≥4	171 (8.7)	68 (13.2)	103 (7.1)	<0.001
upper limbs AIS ≥4	1 (0.1)	1 (0.2)	0 (0.0)	0.26*
lower limbs AIS ≥4	112 (5.7)	21 (4.1)	91 (6.3)	0.06
other AIS ≥4	2 (0.1)	0 (0.0)	2 (0.1)	1*
**ISS **				
15 to 24	457 (23.3)	116 (22.5)	341 (23.7)	0.31
25 to 34	725 (37.0)	207 (40.1)	518 (35.9)	
≥35	471 (24.1)	122 (23.6)	349 (24.2)	

* Fisher Exact Test; AIS, Abbreviated Injury Scale; GCS, Glasgow Coma Scale; GMICU, ground mobile intensive care unit; HMICU, helicopter mobile intensive care unit; ISS, Injury Severity Score; SBP, systolic blood pressure; SpO_2, _pulse oximetry.

The comparison of life-sustaining treatment administered during pre-hospital support is given in Table 3. HMICU patients were treated more aggressively than GMICU patients. Tracheal intubation, administration of crystalloids >1000 ml, treatment with catecholamines and blood product transfusion were more often observed in the HMICU group, whereas colloids or hypertonic saline solution (SSH) were more often used in the GMICU group.

**Table 3:** Pre-hospital life-sustaining treatments according to mode of transport.

		Mode of Transport		
	**all patients** **number (%)** **number = 1,958**	**HMICU** **number (%)** **number = 516**	**GMICU** **number (%)** **number = 1,442**	***P***

Aggressive therapy^a^	287 (14.7)	97 (18.8)	190 (13.2)	0.002
(1)Tracheal intubation	1,050 (53.6)	308 (59.7)	742 (51.5)	0.001
(2) Colloids or SSH	1,074 (54.9)	238 (46.1)	836 (58.0)	<0.001
(3) Crystalloids ≥1000 ml	431 (22.0)	131 (25.4)	300 (20.8)	0.031
(4) Catecholamines	261 (13.3)	93 (18.0)	168 (11.7)	<0.001
(5) Blood products	72 (3.7)	43 (8.3)	29 (2.0)	<0.001
(6) Exsufflation or chest tube	38 (1.9)	14 (2.7)	24 (1.7)	0.14

^a^Aggressive therapy: if three or more of criteria (1) to (6) were present. GMICU, ground mobile intensive care unit; HMICU, helicopter mobile intensive care unit; SSH: hypertonic saline solution.

The surgical procedures performed within 24 hours and until discharge from hospital according to mode of transport are presented in Table 4. Crude mortality before hospital discharge was no different according to pre-hospital mode of transport (88 patients: 17% in HMICU versus 283 patients: 19.6% in GMICU, *P *= 0.20). The risk of death was higher for men, day-time accidents (OR: 0.72, 95% CI 0.55 to 0.95, *P *= 0.018), potentially serious accidents and when there was a suspicion of severe trauma. No link was found between the time to hospital admission and mortality (*P *= 0.96).

**Table 4:** Surgical and hemostatic procedures performed within 24 hours and before discharge from hospital according to mode of transport.

		all patients; number(%) number = 1,958	HMICU; number(%) number = 516	GMICU; number(%) number = 1,442	*P*
**Total surgical procedures **	within 24 hours	909 (46.4%)	252 (48.8%)	657 (45.6%)	0.20
	overall	1,414 (72.2%)	397 (76.9%)	1,017 (70.5%)	0.005
**Head procedure**					
Craniotomy	within 24 hours overall	98 (5.0%) 126 (6.4%)	24 (4.7%) 29 (5.6%)	74 (5.1%) 97 (6.7%)	0.67 0.38
ICP/EVD	within 24 hours overall	- 329 (16,8%)	- 111 (21.5%)	- 218 (15.1%)	<0.001
Face surgery	within 24 hours overall	70 (3.6%) 198 (10.1%)	23 (4.5%) 66 (12.8%)	47 (3.3%) 132 (9.2%)	0.21 0.019
**Thoracic surgery**					
thoracotomy	within 24 hours	36 (1.8%)	7 (1.4%)	29 (2.0%)	0.34
	overall	46 (2.4%)	11 (2.1%)	35 (2.4%)	0.71
chest tube	within 24 hours overall	- 330 (16.9%)	- 95 (18.4%)	- 235 (16.3%)	- 0.27
**Abdominal surgery**	within 24 hours	162 (8.3%)	46 (8.9%)	116 (8.0%)	0.54
	overall	219 (11.2%)	69 (13.4%)	150 (10.4%)	0.067
					
**Spine stabilization**	within 24 hours overall	130 (6.6%) 176 (9.0%)	52 (10.1%) 68 (13.2%)	78 (5.4%) 108 (7.5%)	<0.001 <0.001
**Bone limb fixation**	within 24 hours overall	574 (29.3%) 654 (33.4%)	140 (27.1%) 168 (32.6%)	434 (30.1%) 486 (33.7%)	0.21 0.64
**Wound surgery**	overall	364 (18.6%)	97 (18.8%)	267 (18.5%)	0.89
**Other surgery**	overall	42 (2.2%)	17 (3.3%)	25 (1.7%)	0.036
**Pelvic stabilization**	within 24 hours overall	35 (1.8%) 53 (2.7%)	6 (1.2%) 13 (2.5%)	29 (2.0%) 40 (2.8%)	0.22 0.76
**Angiography**					
alone	within 24 hours overall	- 71 (3.6%)	- 21 (4.1%)	- 50 (3.5%)	- 0.53
with embolization	within 24 hours overall	- 123 (6.3%)	- 32 (6.2%)	- 91 (6.3%)	- 0.93
**Total hemostatic procedures**	Within 24 hours	228 (11.6%)	61 (11.8%)	167 (11.6%)	0.89
	overall	279 (14.3%)	81 (15.7%)	198 (13.7%)	0.28

EVD, external ventricular derivation; GMICU, ground mobile intensive care unit; HMICU, helicopter mobile intensive care unit; ICP, intracanial pressure.

The multivariate analysis for evaluating the association between mode of transport and death before ICU discharge (within 30 days) was performed for 1,817 patients (Table 5). The risk of death was significantly lower (OR: 0.68, 95% CI 0.47 to 0.98, *P *= 0.035) for HMICU compared with GMICU (Table 5, model 1). Increasing age, GCS <14, SBP <90 mmHg, SpO_2 _<90%, suspected trauma severity and aggressive therapy remained significant factors for death, whereas gender and time to hospital admission did not enter the regression model (model 1). The association between the mode of transport and mortality before discharge was unchanged after further adjustment for ISS (model 2), and by overall surgical procedures (model 3). Overall surgical procedures were associated with a reduction in the risk of death (*P *<0.001).

**Table 5:** Association between transport modality and death before ICU discharge (within 30 days) in multivariable analysis

	Model 1			Model 2			Model 3		
	
	OR	95% CI	*P *value	OR	95% CI	*P *value	OR	95% CI	*P *value
**Pre hospital transport **									
GMICU	1	-		1	-		1	-	
HMICU	0.68	0.47-0.98		0.67	0.46-0.97		0.68	0.47-0.99	
**Age **			<0.001			<0.001			<.001
(for 1 year variation)	1.03	1.03-1.04		1.04	1.03-1.04		1.04	1.03-1.04	
**Glasgow Coma Scale **			<0.001			<0.001			<.001
≥14	1	-		1	-		1	-	
8 to 13	2.67	1.67-4.26		2.33	1.44-3.77		2.26	1.39-3.68	
<8	13.37	8.97-9.91		10.84	7.20-16.31		10.11	6.67-15.32	
**Systolic blood pressure (mmHg) **			<0.001			<0.001			<.001
≥110	1	-		1	-		1	-	
90 to 109	1.43	0.95-2.15		1.23	0.81-1.88		1.37	0.89-2.09	
<90	2.79	1.90-4.10		2.5	1.69-3.71		2.49	1.66-3.73	
**SpO_2 _(%) **			0.001			0.001			0.001
>90	1	-		1	-		1	-	
<90	1.83	1.27-2.63		1.83	1.27-2.64		1.89	1.29-2.75	
**Day time**			0.012			0.017			0.021
Day	1	-		1	-		1	-	
Night	0.65	0.46-0.91		0.65	0.46-0.93		0.66	0.46-0.94	
**Aggressive therapy^b^**			0.023			0.066			0.099
No	1	-		1	-		1	-	
Yes	1.54	1.06-2.24		1.44	0.98-2.11		1.39	0.94-2.06	
**Trauma severity**			0.031	not entered		(p>0.10)	not entered		(p>0.10)
No	1	-		-	-		-	-	
Yes	1.4	1.03-1.90		-	-		-	-	
**Injury Severity Score **	not entered					<0.001			<.001
<15	-	-	-	1	-		1	-	
15 to 24	-	-	-	0.77	0.37-1.60		6.54	3.35-12.76	
25 to 34	-	-	-	2.59	1.37-4.89		3.34	1.74-6.43	
≥ 35	-	-	-	4.8	2.51-9.15		0.87	0.41-1.83	
**Surgery (overall)**	not entered			not entered					<.001
No	-	-	-	-	-	-	1	-	
Yes	-	-	-	-	-	-	0.34	0.24-0.48	

**^a^**Analysis performed among 1,817 patients due to missing values for at least one covariate; ^b^therapy defined as aggressive if at least three of six criteria were present: tracheal intubation, colloids or SSH, crystalloids ≥1000 ml, catecholamines, blood products, exsufflation/chest tube. CI, confidence interval; GMICU, ground mobile intensive care unit; HMICU, helicopter mobile intensive care unit; OR, odds ratio; SpO_2_, pulse oximetry; SSH, hypertonic saline solution.

## Discussion

This multicenter cohort study compared HT versus GT of severe blunt trauma patients, both with medical pre-hospital care delivered by MICU, and directly admitted to University hospitals. The probability of death before discharge was lower for helicopter medical transport compared with ground medical transportation. The median hospital admission time was higher for HMICU than for GMICU patients, and the group transported by helicopter received a more aggressive pre-hospital treatment. This association between mode of transport and mortality until discharge was unchanged after further adjustment for ISS. HT was associated with a higher level of medical care and decreased mortality compared with GT.

Helicopters are a costly and limited resource, and their use must take into account their real benefit and risk [22]. Over-triage increases costs and may increase the risk of critical events for transport teams. Under-triage may lead to increased morbidity and mortality in patients who could have benefited from its use.

The present study was specifically performed to investigate the influence of the mode of transport, HT versus GT, on the outcome of injured patients in the context of a pre-hospital medicalized care. The fact that the ground team is identical to the helicopter team (physician, nurse and specially trained pilot/driver) makes it possible to analyze the potential benefit of pre-hospital medical care and its impact on outcome for several categories of patients with as little bias as possible [15]. To limit biases in this comparison, interfacility transfers were excluded and the analysis was restricted to patients directly admitted from the scene to a UnivH trauma center able to provide definitive care for all trauma patients. Similarly, to investigate the real benefit of HT versus GT, patients transported by ground without pre-hospital medical care were excluded.

Patients with an ISS >15 are deemed to require specialized trauma care, while patients with an ISS of 15 or less are considered to have non-life threatening injuries [23,24]. The severity of trauma in the FIRST patients is attested to by the high median ISS and the high percentage of patients with an ISS above 35 (about 25% of all patients). The trauma severity was not different between HMICU and GMICU groups. The percentage of patients with severe spinal injury was higher in the HMICU group than in the GMICU group. The preferred choice of helicopter in cases of spinal injury is probably explained by the higher level of comfort provided by HT.

The median time to hospital admission was longer for HMICU than for GMICU. There is controversy concerning the time spent on the scene and pre-hospital management. The possibility that shortening pre-hospital times improves survival has not yet been demonstrated in studies with appropriate statistical control [25]. Several large studies have demonstrated that, despite a longer transport time associated with HT, trauma patients are more likely to survive and/or to be discharged and allowed to go home after treatment [5,8,26]. This delay may be partly due to a more aggressive therapy observed in HMICU compared with GMICU during the pre-hospital phase. Aeromedical teams usually provide a higher level of care than GMICU teams. Several factors may explain this difference. First, helicopter teams from University hospitals should be more specialized and more highly trained in the care of severely injured patients [27,28]. Helicopter teams usually have a higher level of experience than the GMICU of the nearest hospital for primary care of severe trauma patients. In addition, the decision to administer life-sustaining treatment before HT must be anticipated because of the complexity of performing procedures during the flight.

Few studies have demonstrated a real benefit of high level pre-hospital care. Endotracheal intubation and tension pneumothorax decompression on the scene have been shown to reduce early deaths in trauma [29]. Initial management of patients with severe blunt head trauma requires aggressive volume resuscitation and active drugs to maintain cerebral perfusion pressure, which is directly related to mean arterial blood pressure. The impact of life threatening measures by paramedics on outcome is not clear. The OPALS Major Trauma Study showed that system-wide implementation of full advanced life support programs for paramedics does not decrease mortality or morbidity in major trauma patients [30]. Therefore, the major benefit of HMICU seems to be the high rate of early intervention by medical air teams, the quality of life-sustaining treatment and decision-making and a more aggressive on-site approach [10,29,31]. A better outcome with this strategy has been described for severe traumatic brain injury [11].

Our results showed that the need for emergency surgical procedures, and overall head surgical procedures until discharge from hospital, was higher in the HMICU group than in the GMICU group. Although crude mortality until hospital discharge was no different according to pre-hospital mode of transport, the probability of death before hospital discharge was lower for the HMICU group in the multivariate analysis, whatever the model considered. HT seems to act as an accelerator of care within the hospital, probably with a higher quality of care by the team in charge of the patient on arrival at the trauma center.

Our study must be interpreted with caution because of its methodological limitations. The FIRST study is observational so that no causal inference can be drawn from our findings. The decision to dispatch a helicopter is based on multiple factors, and there is currently no standardization of these criteria in France. There may thus be differences between the various centers in how this mode of transport is chosen. Hospitals participating in the FIRST study were UnivH. These trauma centers were able to provide definitive care, but the level of care for each patient may have been different in the absence of procedures validating the ability of staff to manage all trauma patients. However, to take into account potential between-center differences in patient management, all analyses were stratified by center. Another weakness was our limited ability to distinguish between urban and rural intervention areas which may influence the use of HT. Furthermore, the distance from the scene of the accident to the trauma center was not recorded, and we were unable to distinguish the time dedicated to transport and the time for performing life-sustaining treatment by the team. Despite our careful adjustment strategy, we cannot exclude residual confounding due to non-measured factors such as comorbidities and detailed schedule of pre-hospital care. A further limitation of this study was the lack of information on patient quality of life. Helicopters may reduce mortality rates, leaving patients disabled and with a lower quality of life in the long-term. Finally, our study was conducted only in patients who were managed by MICU prior to hospital admission; this is not the case for all pre-hospital systems, making it difficult to generalize about our results.

## Conclusions

This original comparison of helicopter utilization in the pre-hospital context shows that severe trauma patients transported by helicopter medical teams received more aggressive therapy during the pre-hospital phase than patients transported by ground medical teams. Their probability of death was decreased with HT after adjustment for initial physiological status and trauma severity compared with patients transported by GMICU. It remains unclear whether or not this benefit can be attributed exclusively to the high level of pre-hospital care. More aggressive therapy during the pre-hospital phase and more overall surgical procedures during the hospital phase cannot alone explain this benefit. Furthermore, more detailed analysis of the pattern of pre-hospital care is needed to explore other factors that may help to explain this benefit. Finally, HT is more expensive than GT and a cost effectiveness analysis may be the most appropriate step for further studies.

## Key messages

• HMICU patients had a higher median time frame before hospital admission

• HMICU patients were more intensively treated in the pre-hospital phase

• After adjustment for initial status, the risk of death was significantly lower for HMICU compared with GMICU

• This study suggests a beneficial impact of HT on mortality in severe blunt trauma

• Whether this association could be due to better management in the pre-hospital phase needs to be more thoroughly assessed.

## Abbreviations

AIS: Abbreviated Injury Scale; CI: confidence interval; EVD: external ventricular derivation; GCS. Glasgow Coma Scale; GMICU: Ground Mobile Intensive Care Unit; GT: ground transport; HMICU: Helicopter Mobile Intensive Care Unit; HT: helicopter transport; ICP: intracranial pressure; IQR: interquartile range; ISS: Injury Severity Score; OR: odds ratio; SBP: systolic blood pressure; SD: standard deviation; SpO_2, _pulse oximetry; UnivH: university hospital.

## Competing interests

The authors declare that they have no competing interests.

## Authors' contributions

TD was involved in the analysis and interpretation of data and drafted the manuscript. JMY was involved in the study design, in the acquisition of data and in the final revision of the manuscript. HC, CJ, MR, BV and CM participated in the acquisition and interpretation of data and in the final revision of the manuscript. CBK was responsible for the logistic coordination of the study, was involved in the design of the study, in statistical analysis and interpretation of data and helped to draft the manuscript. MF initiated and coordinated the study and was involved at all steps of the study. All authors read and approved the final manuscript.

## References

[B1] Kreis DJ Jr Plasencia G Augenstein D Davis JH Echenique M Vopal J Byers P Gomez G Preventable trauma deaths: Dade County, Florida J Trauma 1986 16 649 654 10.1097/00005373-198607000-00010 3723641

[B2] Gruen RL Jurkovich GJ McIntyre LK Foy HM Maier RV Patterns of errors contributing to trauma mortality: lessons from 2,594 deaths Ann Surg 2006 16 371 380 16926563 10.1097/01.sla.0000234655.83517.56 PMC1856538

[B3] MacKenzie EJ Rivara FP Jurkovich GJ Nathens AB Frey KP Egleston BL Salkever DS Scharfstein DO A national evaluation of the effect of trauma-center care on mortality N Engl J Med 2006 16 366 378 10.1056/NEJMsa052049 16436768

[B4] Bledsoe BE Wesley AK Eckstein M Dunn TM O'Keefe MF Helicopter scene transport of trauma patients with nonlife-threatening injuries: a meta-analysis J Trauma 2006 16 1257 1265 10.1097/01.ta.0000196489.19928.c0 16766969

[B5] Brown JB Stassen NA Bankey PE Sangosanya AT Cheng JD Gestring ML Helicopters and the civilian trauma system: national utilization patterns demonstrate improved outcomes after traumatic injury J Trauma 2010 16 1030 1034 10.1097/TA.0b013e3181f6f450 21068607

[B6] Bulger EM Guffey D Guyette FX MacDonald RD Brasel K Kerby JD Minei JP Warden C Rizoli S Morrison LJ Nichol G Resuscitation Outcomes Consortium Investigators Impact of prehospital mode of transport after severe injury: a multicenter evaluation from the Resuscitation Outcomes Consortium J Trauma Acute Care Surg 2012 16 567 573 22491538 10.1097/TA.0b013e31824baddf PMC3495608

[B7] Brown JB Stassen NA Bankey PE Sangosanya AT Cheng JD Gestring ML Helicopters improve survival in seriously injured patients requiring interfacility transfer for definitive care J Trauma 2011 16 310 314 10.1097/TA.0b013e3182032b4f 21307726

[B8] Galvagno SM Jr Haut ER Zafar SN Millin MG Efron DT Koenig GJ Jr Baker SP Bowman SM Pronovost PJ Haider AH Association between helicopter vs ground emergency medical services and survival for adults with major trauma JAMA 2012 16 1602 1610 10.1001/jama.2012.467 22511688 PMC3684156

[B9] Isakov A Urgent air-medical transport: right patient, place and time CMAJ 2009 16 569 570 10.1503/cmaj.091258 19752100 PMC2764747

[B10] Biewener A Aschenbrenner U Rammelt S Grass R Zwipp H Impact of helicopter transport and hospital level on mortality of polytrauma patients J Trauma 2004 16 94 98 10.1097/01.TA.0000061883.92194.50 14749573

[B11] Berlot G La Fata C Bacer B Biancardi B Viviani M Lucangelo U Gobbato P Torelli L Carchietti E Trillò G Daniele M Rinaldi A Influence of prehospital treatment on the outcome of patients with severe blunt traumatic brain injury: a single-centre study Eur J Emerg Med 2009 16 312 317 10.1097/MEJ.0b013e32832d3aa1 19491690

[B12] Taylor CB Stevenson M Jan S Middleton PM Fitzharris M Myburgh JA A systematic review of the costs and benefits of helicopter emergency medical services Injury 2010 16 10 20 10.1016/j.injury.2009.09.030 19853251

[B13] Yeguiayan JM Garrigue D Binquet C Jacquot C Duranteau J Martin C Rayeh F Riou B Bonithon-Kopp C Freysz M French Intensive Care Recorded In Severe Trauma Study Group. Medical pre-hospital management reduces mortality in severe blunt trauma: a prospective epidemiological study Crit Care 2011 16 R34 10.1186/cc9982 21251331 PMC3222071

[B14] Spaulding CM Joly LM Rosenberg A Monchi M Weber SN Dhainaut JF Carli P Immediate coronary angiography in survivors of out-of-hospital cardiac arrest N Engl J Med 1997 16 1629 1633 10.1056/NEJM199706053362302 9171064

[B15] Nathens AB Brunet FP Maier RV Development of trauma systems and effect on outcomes after injury Lancet 2004 16 1794 1801 10.1016/S0140-6736(04)16307-1 15172780

[B16] Ricard-Hibon A Marty J Management of severe head-injured patients in the first 24 hours. Resuscitation and initial diagnostic strategy Ann Fr Anesth Réanim 2000 16 286 295 10836116 10.1016/s0750-7658(99)00149-5

[B17] Sartorius D Le Manach Y David JS Rancurel E Smail N Thicoïpé M Wiel E Ricard-Hibon A Berthier F Gueugniaud PY Riou B Mechanism, glasgow coma scale, age, and arterial pressure (MGAP): a new simple prehospital triage score to predict mortality in trauma patients Crit Care Med 2010 16 831 837 10.1097/CCM.0b013e3181cc4a67 20068467

[B18] Freysz M Yeguiayan JM Evaluation of the severity and monitoring of early complications in multitrauma Rev Prat 2007 16 441 452 17455747

[B19] Champion HR Copes WS Sacco WJ Lawnick MM Bain LW Gann DS Gennarelli T Mackenzie E Schwaitzberg S A new characterization of injury severity J Trauma 1990 16 539 545 10.1097/00005373-199005000-00003 2342136

[B20] Baxt W Jones J Fortlage D The trauma triage rule: a new resource-based approach to the prehospital identification of major trauma victims Ann Emerg Med 1991 16 1404 1406 10.1016/s0196-0644(05)82608-3 2240753

[B21] American College of Surgeon's Committee on Trauma, ASCOT Resources for Optimal Care of the Injured Patient 1999 Chicago, American College of Surgeons

[B22] Singh JM MacDonald RD Bronskill SE Schull MJ Incidence and predictors of critical events during urgent air-medical transport CMAJ 2009 16 579 584 10.1503/cmaj.080886 19752105 PMC2764752

[B23] Baker SP O'Neill B Haddon W Jr Long WB The Injury Severity Score: a method for describing patients with multiple injuries and evaluating emergency care J Trauma 1974 16 187 196 10.1097/00005373-197403000-00001 4814394

[B24] Kane G Englehardt R Celentano J Koenig W Yamanaka J McKinney P Brewer M Fife D Empirical development and evaluation of pre-hospital trauma triage instruments J Trauma 1985 16 482 489 10.1097/00005373-198506000-00002 4009748

[B25] Ringburg AN Spanjersberg WR Frankema SP Steyerberg EW Patka P Schipper IB Helicopter emergency medical services (HEMS): impact on on-scene times J Trauma 2007 16 258 262 10.1097/01.ta.0000240449.23201.57 17693821

[B26] Sullivent EE Faul M Wald MM Reduced mortality in injured adults transported by helicopter emergency medical services Prehosp Emerg Care 2011 16 295 302 10.3109/10903127.2011.569849 21524205

[B27] Baxt WG Moody P The impact of rotorcraft aeromedical emergency care service on trauma mortality JAMA 1983 16 3047 3051 10.1001/jama.1983.03330460029027 6854826

[B28] Boyd CR Corse KM Campbell RC Emergency interhospital transport of the major trauma patient: air versus ground J Trauma 1989 16 789 794 10.1097/00005373-198906000-00015 2738976

[B29] Schmidt U Frame SB Nerlich ML Rowe DW Enderson BL Maull KI Tscherne H On-scene helicopter transport of patients with multiple injuries-comparison of a German and an American system J Trauma 1992 16 548 53 10.1097/00005373-199210000-00010 1433401

[B30] Stiell IG Nesbitt LP Pickett W Munkley D Spaite DW Banek J Field B Luinstra-Toohey L Maloney J Dreyer J Lyver M Campeau T Wells GA OPALS Study Group The OPALS Major Trauma Study: impact of advanced life-support on survival and morbidity CMAJ 2008 16 1141 1152 10.1503/cmaj.071154 18427089 PMC2292763

[B31] Roberts K Blethyn K Foreman M Bleetman A Influence of air ambulance doctors on on-scene times, clinical interventions, decision-making and independent paramedic practice Emerg Med J 2009 16 128 134 10.1136/emj.2008.059899 19164630

